# Transcutaneous CO_2_ Measurement in an Adult Long-Term Ventilation (LTV) Service

**DOI:** 10.3390/jcm14124137

**Published:** 2025-06-11

**Authors:** Wei Hann Ong, Peter Ireland, Ching Khai Ho, Ross Fowkes, Yamuna Madhu, Richard Davidson, Katie Kaiser, Kathy George, Jane Rodger, Alison Armstrong, Ben Messer, Hilary Tedd, Nicholas Lane, Anthony De Soyza

**Affiliations:** 1 North East Assisted Ventilation Service, Royal Victoria Infirmary, The Newcastle upon Tyne Hospitals NHS Foundation Trust, Newcastle upon Tyne NE1 4LP, UK; peter.ireland2@nhs.net (P.I.); chingkhai.ho@nhs.net (C.K.H.); rossfowkes@nhs.net (R.F.); yamuna.madhu1@nhs.net (Y.M.); richard.davidson7@nhs.net (R.D.); katie.kaiser@nhs.net (K.K.); kathy.george1@nhs.net (K.G.); jane.rodger3@nhs.net (J.R.); alison.armstrong1@nhs.net (A.A.); ben.messer@nhs.net (B.M.); hilary.tedd1@nhs.net (H.T.); nicholas.lane@nhs.net (N.L.); 2 Translational and Clinical Research Institute, Faculty of Medical Sciences, Newcastle University, Newcastle upon Tyne NE1 7RU, UK; 3 Population Health Sciences Institute, Newcastle University, Ridley 1 Building, Newcastle upon Tyne NE1 4LP, UK

**Keywords:** transcutaneous carbon dioxide, ventilatory failure, non-invasive ventilation, assisted ventilation

## Abstract

**Background**: Transcutaneous CO_2_ (TcCO_2_) measurement is widely used in the diagnosis and monitoring of ventilatory failure. Robust data on the success rates of measurement is scant. We aimed to discern the factors affecting the success rate of TcCO_2_ measurement in a regional LTV service. **Methods**: Patients undergoing TcCO_2_ measurement between October 2019 and January 2022 were identified retrospectively. Notes were analysed for basic demographics, indications for TcCO_2_ measurement, measurement outcome, device used (Radiometer TCM5 or Sentec, based on availability), setup (self, carer, or clinician), inpatient or domiciliary study, and number of TcCO_2_ measurement attempts occurred. Statistical comparisons were made by Fisher’s exact test. **Results**: We identified 435 recording events on 288 patients, mean age of 53, and 56% were males. A total of 189 (66%) had a neuromuscular disorder (NMD). The commonest indications for TcCO_2_ measurement were ‘assessing ventilatory failure’ (43%) in treatment-naïve patients and ‘adequacy of ventilation therapy due to persistent symptoms’ (26%) in those established on LTV. Over 80% of our recording events were applied by patients or their carers. Overall, TCM5 devices had statistically higher successful recording rates (197/268, 73.5%) than Sentec (100/165, 60.6%) [*p* = 0.0056]. In domiciliary studies, TCM5’s success rate of 187/253 (73.9%) versus Sentec’s 94/154 (61.0%) was significantly better [*p* = 0.0079]. The success rate of each measurement attempt ranged between 62.9 and 67.0%, with up to three attempts on each subject. **Conclusions**: Home TcCO_2_ is helpful in managing those with respiratory failure. Repeating tests after initial failure of recording is worthwhile. There may be differences in performance across devices which warrants further study.

## Simple Summary

Transcutaneous carbon dioxide (TcCO_2_) measurement is a non-invasive method of real-time CO_2_ monitoring, increasingly utilised in inpatient and domiciliary settings. We assessed the factors affecting success rate of TcCO_2_ measurement. This study reinforced its usefulness in diverse clinical settings, with the caveat that there is the potential for differences in performance with different devices.

## 1. Introduction

Individuals with chronic respiratory insufficiency often require long-term ventilation (LTV) at home to control ventilatory failure. Continuous monitoring of carbon dioxide (CO_2_) levels is helpful in diagnosing ventilatory failure, as well as ascertaining appropriate ventilation settings to minimise associated complications. Conventionally, this is achieved via arterial blood gas (ABG) sampling to measure the PaCO_2_. However, ABG sampling provides only a static time point measurement. Subjecting patients to repeated invasive procedures is arguably impractical, especially with its considerable failure rate and recognised complications such as pain, infection, and nerve and tissue damage [1,2].

Transcutaneous carbon dioxide (TcCO_2_) measurement has emerged as a non-invasive method for real-time monitoring of CO_2_ levels. These devices were developed primarily for inpatient settings, but with time their use in domiciliary settings is becoming very common. In our unit, two commonly used TcCO_2_ monitoring systems, Sentec (Switzerland) and Radiometer TCM5 (Copenhagen, Denmark), have shown promise across clinical settings.

The primary objective of this study was to assess the factors affecting the success rate of TcCO_2_ measurement in the individuals referred to our LTV service for assessment of ventilatory failure, or in those already under our care and being treated with non-invasive ventilation (NIV). These include the “real world” outcomes of applying Sentec and Radiometer TCM5 TcCO_2_ monitoring systems; we studied different approaches of setup, i.e., self, clinician, or carer setup, as well as the monitoring environment, be it inpatient or domiciliary.

## 2. Methods

Our retrospective observational study was conducted in adults aged 18 or greater, requiring or being considered for long-term home ventilation. The indication for monitoring of transcutaneous carbon dioxide was therefore to define the presence of respiratory failure or to monitor responses to non-invasive ventilation. Local governance approvals were obtained and, as an audit, the study did not require individual patient consent. The data capture period study duration was between October 2019 to January 2022, a total of 28 months.

Our setting was the North East Assisted Ventilation Service (NEAVS), Northeast England, UK. We provide services to two tertiary care NHS Trusts and multiple secondary care sites managing long-term neuromuscular or primary respiratory diseases. Inclusion criteria comprised adults being referred for assessment of ventilatory failure, or patients established on home ventilation and deemed to require TcCO_2_ measurement by their treating clinician.

**Figure 1:**
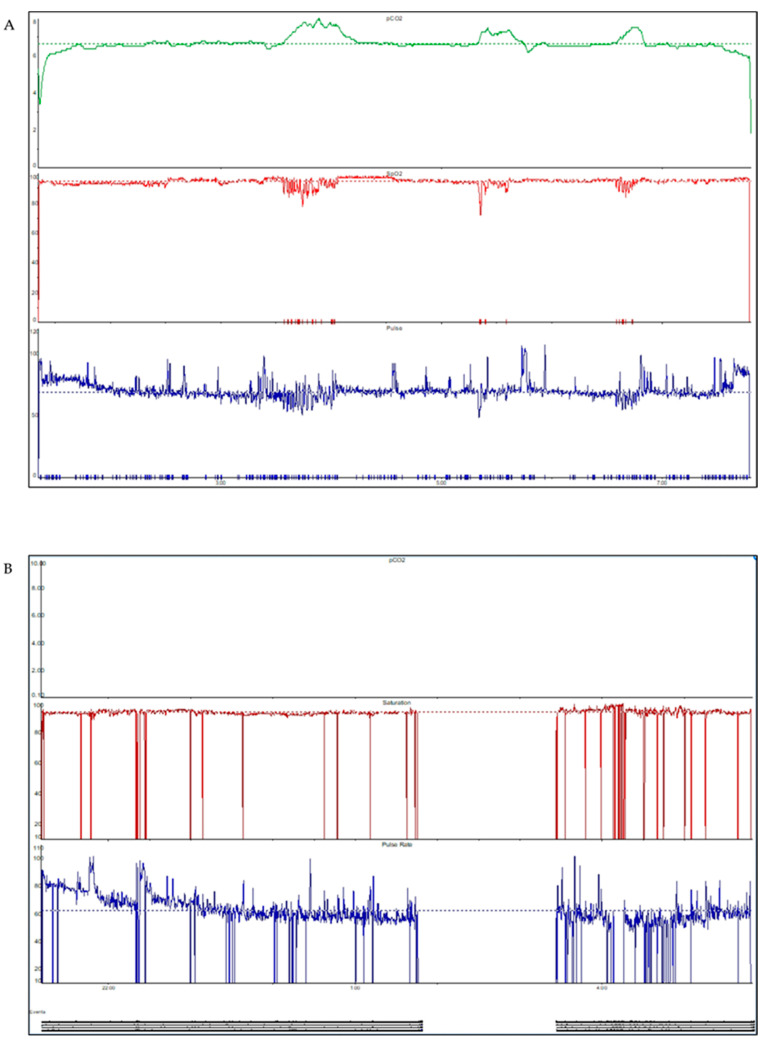
Examples of pCO_2_ traces on TcCO_2_ measurement: (**A**) good pCO_2_ traces; (**B**) no pCO_2_ traces.

We undertook retrospective data collection from patients’ electronic clinical notes. We collected participants’ demographic data, including their primary condition predisposing to ventilatory failure (e.g., neuromuscular disease, structural chest wall deformity, primary respiratory disease such as chronic obstructive pulmonary disease (COPD) and obesity hypoventilation syndrome, and spinal cord/traumatic brain injury), setting for the test (inpatient vs. domiciliary), who applied TcCO_2_ monitor probes (e.g., clinicians, patient, or patients’ care team or family), and number of attempts at TcCO_2_ measurement before a successful result was obtained. Successful measurement was defined as the recording of an adequate dataset or pCO_2_ traces (see Figure 1) resulting in a treatment decision.

We defined 5 indications for undertaking TcCO_2_ measurement:

(1) Establishing the presence of diaphragmatic weakness or ventilatory failure;
(2) Helping to distinguish between obstructive sleep apnoea (OSA) and obesity-related respiratory failure;
(3) Establishing the adequacy of ventilation (either routinely or due to patient-reported symptoms suggesting inadequate ventilation);
(4) Establishing the ongoing need for NIV in patients who are already on long-term NIV;
(5) Pre-procedural monitoring in high-risk individuals.

In addition to establishing the above category, we recorded if TcCO_2_ monitoring was performed whilst the patient was using a ventilator or not.

All participants underwent TcCO_2_ measurements using either a Sentec or Radiometer TCM5 device based on local machine availability at the time (we have 3 TCM5 and 4 Sentec devices in our LTV service and both manufacturers’ machines were available for use during the period of study). The TcCO_2_ probes were applied according to the manufacturer’s instructions. Continuous measurements were taken overnight. Patients received either inpatient or domiciliary measurement depending on clinical circumstances. Home or non-hospital care facility-based recordings were conducted after patients or carers were either trained or provided with instructions, and then recording was undertaken at home (defined as domiciliary non-clinician-applied TcCO_2_); or in some instances clinical staff applied the TcCO_2_ monitoring probes at home (domiciliary clinician-applied TcCO_2_); see Table 1. In our routine practice, a healthcare assistant from the team typically visits the patient’s residence to provide training to the patient or caregiver on the use of the monitoring device. In addition to in-person instruction, an instructional guide is supplied to support independent application. As the healthcare assistant service operates during standard office hours, direct application of the device by staff is not routinely feasible.

Subsequent statistical comparisons were completed using Fisher’s exact test.

## 3. Results

288 participants were included with 435 recorded events. The mean age was 53 years (SD 19.9) with 56% males. The commonest indications for TcCO_2_ measurement in our LTV service were ‘assessment of diaphragmatic weakness or hypoventilation’ (N = 125; 43%) in those naïve to ventilation, and ‘assessing adequacy of home ventilation therapy due to persistent symptoms’ in the cohort who were already established on non-invasive ventilatory treatment (N = 74; 26%).

In 189 (66%) patients the aetiology was a neuromuscular disorder. This included 61 (21%) patients with motor neurone disease (MND), 17 (6%) with Duchenne muscular dystrophy (DMD), and 111 (39%) other neuromuscular disorders/muscular dystrophies (e.g., Becker muscular dystrophy, myotonic dystrophies, congenital muscular dystrophies, etc). Obesity hypoventilation syndrome (OHS) and COPD accounted for 32 (11%) and 12 (4%) of subjects, respectively. The main aims of TcCO_2_ recordings were to investigate possible ventilatory failure (43%) and to assess the adequacy of ventilation on NIV (routine 17% and due to symptoms of NIV 26%).

The overall success rate of TcCO_2_ measurement was 68.3%. Of 435 recording events, 267 (61.4%) used the Radiometer TCM5 device, and 166 (38.1%) used the Sentec device (with missing documentation of device for two events). Most were performed in a domiciliary setting (N = 409, 94%) with 205 being set up by patients, 152 set up by carers, and the remaining 52 not specified. All 26 inpatient recording events were setup by clinicians, either from the long-term ventilation service or inpatient care team. A total of 307 recording events were first attempts (71%; 2nd attempt 20%; and 9% were 3rd attempt); See Table 2.

We investigated several factors that may affect TcCO_2_ measurement independently (Table 3): type of devices (Radiometer TCM5 and Sentec), settings (inpatient versus domiciliary), setup (patient, carer, or clinician), and number of attempts, as well as device delivery and probe application training.

Overall, the TCM5 devices had higher successful recording rates (73.5%) than the Sentec devices (60.6%) [*p* = 0.0056]. TcCO_2_ monitoring in domiciliary setups yield a higher success rate (69.1%) than inpatient setups (57.7%) although the sample sizes of inpatient studies were small. In terms of setup type, success rates were higher when clinicians (73.1%) or carers (71.7%) performed the setup, whilst self-setup was less successful (64.4%). The group receiving devices via courier with no additional training, but who had prior user experience still had a relatively high success rate (70.0%), close to that of fully trained clinician-delivered setups (67.6%). However, looking at the different devices used in each of these factors, the results were all in favour of the TCM5 device; see Table 4. For example, in domiciliary studies, TCM5’s success rate at 187/253 (73.9%) versus Sentec’s 94/154 (61.0%) was statistically significantly better [*p* = 0.0079]. Inpatient studies have also trended correspondingly but failed to reach significance, likely due to small sample sizes. When comparing the setup, in the non-clinician setup group, TCM5s achieved a 171/218 (78.1%) success rate, compared to Sentec’s 80/139 (57.6%) [*p* < 0.001]. There was a similar finding in the clinician setup group although this was not statistically significant (sample size, n = 25).

Looking at overall transcutaneous CO_2_ measurement events within the study period, the success rate was 67% on the first attempt, 69.4% on the second, and 75.6% after three or more attempts (see Table 5). We recognised that this is not a true reflection of the success rate for each attempt because of the non-inclusion of uncaptured attempts outside the observed period.

We carried out further analysis to explore this. Amongst individuals who did not achieve success on the initial attempt (N = 106), 62 underwent a second attempt, with 39 successful outcomes. Of the remaining 23, 11 proceeded to a third attempt, with 7 yielding successes.

**Figure 2:**
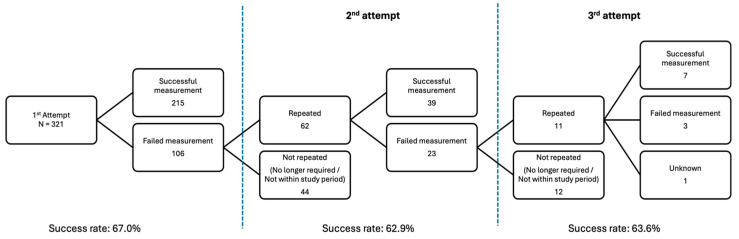
Success rate of TcCO_2_ measurement on repeated attempts.

Cumulatively, the success rate improved steadily from 67% after the first attempt to 79% after the second, and eventually to 81% after the third attempt. When analysing the success rate of each subsequent attempt individually, the rates ranged between 62.9% and 67% (see Figure 2).

Upon successful TcCO_2_ measurement, 110/297 (37%) progressed to new ventilation setup or required a change in established ventilator settings; most notably in patients diagnosed with NMDs (83/203, 41%).

## 4. Discussion

Long-term non-invasive ventilation in a domiciliary setting is becoming more prevalent due to significant expansion in both accepted indications and demand. Decision-making on commencing ventilation beyond a single static point of care ABG assessment is frequently required. For individuals already on ventilation, either recently commenced or those established but with emergent or worsening symptoms of hypercapnia, TcCO_2_ is useful to reassess the adequacy of ventilator settings.

Prior diagnostic accuracy studies of other non-invasive monitoring modalities in ventilatory failure concluded that nocturnal peripheral oxygen saturation (SpO_2_) and daytime ABG failed to accurately detect hypoventilation and emphasised the importance of nocturnal monitoring of CO_2_ [3]. TcCO_2_ monitoring provides real-time data that can be diagnostic of periodic nocturnal desaturation associated with diaphragmatic weakness, particularly in REM-related disorders. Whilst not yet widely adopted in community settings, we believe that our study will demonstrate that TcCO_2_ measurement complements existing investigation modalities, such as overnight oximetry, polysomnography, blood gas analysis, and pulmonary function tests (PFTs), by enhancing the ability to detect and quantify sleep-related hypoventilation. Each of these tests contributes to increasing the pre-test probability of clinically significant overnight episodic desaturation, and TcCO_2_ monitoring offers a non-invasive option to strengthen diagnostic confidence.

There is also emerging evidence that overnight capnography has been shown to predict poor outcomes in ventilated patients with Duchenne muscular dystrophy (DMD) [4]. Our study contributes to this growing body of evidence, supporting the integration of TcCO_2_ monitoring into a broader diagnostic framework for patients with suspected nocturnal hypoventilation. Furthermore, Georges et al. reported in those already on ventilation that the most effective strategy was TcCO_2_ measurement supplemented with the use of ventilator software assessment [5].

Current ERS guidelines for long-term ventilation in COPD recommend monitoring of CO_2_ levels. They state that TcCO_2_ monitoring is commonplace in clinical care. However, they do not comment on how frequently to monitor CO_2_, nor advise on how this should be conducted [6,7].

Previous guidelines note that TcCO_2_ devices are safe and should be applied for monitoring CO_2_ but require application by experienced trained staff [8]. More recently, Malone et al. reported that mechanically ventilated inpatients where the monitors were applied by trained staff using TcCO_2_ measurement had a 90% success rate in attaining satisfactory traces [9]. TcCO_2_ monitoring serves as a valuable modality for the detection of nocturnal hypercapnia, with the goal of identifying respiratory insufficiency prior to the development of daytime hypercapnia. The onset of daytime hypercapnia is associated with a markedly poor prognosis, with reported median survival times of 9.7 months in patients with Duchenne muscular dystrophy (DMD) [10] and as little as 11 days in patients with motor neurone disease who retain good bulbar function [11].

Our data suggests that both Sentec and Radiometer TCM5 TcCO_2_ monitoring systems are useful even when applied at home by non-clinicians. However, the observed differences between the two systems raises important considerations for clinical practice. The higher failure rate for recording TcCO_2_ values by Sentec may have multiple underlying reasons reflecting the real-life setting and include differences in preparation and application of the monitors. After our initial data collection, we undertook a period of retraining, working with the manufactures of the Sentec device. Our learning points were related to electrode placement and positioning, as well as refining skin preparation techniques. A recent meta-analysis of 2817 patients concluded that TcCO_2_ sensors may be temperature and positionally sensitive and recommends that they should preferentially be applied to the earlobe [12].

Additionally, increasing the number of measurements attempts during TcCO_2_ monitoring has been shown to improve the overall success rate by approximately two thirds on each further attempt in our study. This aligns with our learning points, where previous studies also indicated that TcCO_2_ accuracy is sensitive to factors such as skin perfusion and sensor positioning [10] and multiple attempts help to overcome these challenges, leading to more reliable and consistent results. This emphasises that repeated attempts help refine the procedure for optimal accuracy.

This study has several limitations that should be acknowledged. Firstly, ABG analysis was not routinely performed concurrently with TcCO_2_ measurements, limiting the ability to directly validate TcCO_2_ values within this cohort. Nevertheless, the correlation between TcCO_2_ and PaCO_2_ has been well-established in previous studies, including the study by Conway et al. [12] which supports the reliability of TcCO_2_ as a surrogate measure.

Clinician-led device setup was infrequent, occurring primarily during inpatient assessments and a small number of domiciliary studies. Due to the small number of these instances, the rationale for clinician involvement was not systematically recorded, which restricts our ability to assess the impact of this variable on setup success.

A further limitation is the absence of outcome data in relation to the use of TcCO_2_ monitoring. Although measurement frequently influenced clinical decision-making regarding the initiation of ventilation and adjustment of ventilator settings, we were unable to quantify its effect on patient outcomes, as such data were not retrievable from our electronic health records.

In addition, while TcCO_2_ monitoring is useful in detecting nocturnal hypercapnia, it does not distinguish between underlying pathophysiological mechanisms such as apnoeas, hypopnoeas, ineffective respiratory efforts, mask leaks, or ventilator asynchrony. Although our findings suggest the utility of TcCO_2_ monitoring across the five clinical contexts described in our methodology, further research is required to define its precise role within the broader diagnostic pathway.

From a health economics perspective, although home mechanical ventilation (HMV) services in the UK are generally funded through block contracts, where individual investigations are not charged per use, the costs associated with TcCO_2_ monitoring remain substantial. These include an estimated GBP 10,000 per device, GBP 4000 per sensor, and a small cost for consumables and disposables such as gel and adhesive stickers, potentially limiting widespread implementation.

Whilst TcCO_2_ monitoring primarily focuses on measuring CO_2_ levels, other parameters such as oxygen saturation and qualitative analysis of CO_2_ traces can also provide valuable insights. These additional metrics may help improve the overall understanding of a patient’s respiratory status. However, in our study, we were unable to draw definitive conclusions regarding the significance of these additional factors, and further research is needed to explore their potential clinical relevance. Future studies should consider diagnostic accuracy, such as by comparing different devices simultaneously in the same patients. However, this may be challenging as sicker patients may be unlikely to engage in repeated testing.

## 5. Conclusions

We show that the outcome of TcCO_2_ measurement, even when applied by patients or carers, is a valuable tool for continuous, non-invasive CO_2_ measurement in outpatient settings, but different devices may have different performance. TcCO_2_ monitors support clinical decision making and repeat attempts are useful after initial failure.

## Figures and Tables

**Table 1:** Basic demographics and indications for TcCO_2_ measurement.

	Number of Subjects, N	%
**Gender**		
Male	161	56
**Age (years)**		
18–40	90	31
41–64	92	32
>65	106	37
Mean (SD)	53 (19.9)	
**Underlying aetiology**		
Chronic obstructive pulmonary disease (COPD)	12	4
Obesity-related respiratory failure	32	11
Motor neurone disease (MND)	61	21
Duchenne muscular dystrophy (DMD)	17	6
Other neuromuscular diseases	111	39
Spinal cord injury	15	5
Congenital/traumatic brain injury (TBI)	6	2
Chest wall deformity	26	9
Other airway disease	8	3
**Indication for TcCO_2_ measurement**		
Assessment of diaphragmatic weakness/hypoventilation	125	43
Distinguishing OSA and obesity-related respiratory failure	22	7
Adequacy of ventilation (routine)	48	17
Adequacy of ventilation due to persistent symptoms	74	26
Pre-procedural	2	1
Assessing ongoing need to continue on NIV	17	6

**Table 2:** Factors identified that may affect TcCO_2_ measurement.

Transcutaneous CO_2_ Measurement (Total Recording Events, N = 435)	Number of Events, N	%
**Successful**	297	68.3
**Failed**	138	31.7
**Monitoring device**		
Radiometer TCM5	267	61.4
Sentec	166	38.1
Unknown	2	0.5
**Measurement settings**		
Inpatient	26	6
Domiciliary	409	94
**Setup**		
Patient self-setup	205	47
Carer	152	35
Clinician	26	6
Unknown	52	12
**Device delivery and application training**		
Device and application both delivered by clinician	260	60
Device delivered via courier, with no training provided (patient/carer has previous user experience)	80	18
Device set up by clinician, with no training delivered	19	5
Information unavailable	76	17
**Number of attempt(s)**		
One	307	70.5
Two	87	20
Three or more	41	9.5

**Table 3:** Outcomes of transcutaneous CO_2_ measurement in different types of device, various settings, setups, and device delivery with application training.

** Type of device**	** Successful measurement,** ** N (%)**	** Failed measurement,** ** N (%)**	** Total**
*TCM5*	197 (73.5%)	71 (26.5%)	267
*Sentec*	100 (60.6%)	65 (39.4%)	166
*Unknown*	1 (50%)	1 (50%)	2
** Setting**	**Successful measurement,** **N (%)**	**Failed measurement,** **N (%)**	**Total**
*Inpatient*	15 (57.7%)	11 (42.3%)	26
*Domiciliary*	283 (69.1%)	126 (30.9%)	409
** Setup**	**Successful measurement,** **N (%)**	**Failed measurement,** **N (%)**	**Total**
*Self setup*	132 (64.4%)	73 (35.6%)	205
*Carer setup*	109 (71.7%)	43 (28.3%)	152
*Clinician setup*	19 (73.1%)	7 (26.9%)	26
*Unknown*	37 (71.2%)	15 (28.8%)	52
** Device delivery/Application training**	**Successful measurement,** **N (%)**	**Failed measurement,** **N (%)**	**Total**
*Clinician delivered/Application training delivered*	176 (67.6%)	84 (32.4%)	260
*Device delivered via courier/No application training (has previous user experience)*	56 (70.0%)	24 (30.0%)	80
*Device set up by clinician/No application training delivered*	19 (73.1%)	7 (26.9%)	26
*Information unavailable*	46 (65.3%)	23 (34.7%)	69

**Table 4:** Outcomes of transcutaneous CO_2_ measurement in various settings and setups, in comparison to types of devices used.

** Overall,** N = 433			
	**Successful measurement, N (%)**	**Failed measurement, N (%)**	*p* = 0.0056
*TCM5*	197 (73.5%)	71 (26.5%)	
*Sentec*	100 (60.6%)	65 (39.4%)	
** Inpatient Study,** N = 26			
	**Successful measurement, N (%)**	**Failed measurement, N (%)**	*p* = 0.6922
*TCM5*	9 (64.3%)	5 (35.7%)	
*Sentec*	6 (50%)	6 (50%)	
** Domiciliary Study,** N = 407			
	**Successful measurement, N (%)**	**Failed measurement, N (%)**	*p* = 0.0079
*TCM5*	187 (73.9%)	66 (26.1%)	
*Sentec*	94 (61.0%)	60 (39.0%)	
** Non-clinician setup (self/carer setup),** N = 358			
	**Successful measurement, N (%)**	**Failed measurement, N (%)**	*p* < 0.0001
*TCM5*	171 (78.1%)	48 (21.9%)	
*Sentec*	80 (57.6%)	59 (42.4%)	
** Clinician setup,** N = 25			
	**Successful measurement, N (%)**	**Failed measurement, N (%)**	*p* = 0.6729
*TCM5*	12 (75.0%)	4 (25.0%)	
*Sentec*	6 (66.7%)	3 (33.3%)	

**Table 5:** Successful rate of transcutaneous CO_2_ measurement corresponding with the number of attempts.

Number of Attempt (s)	Successful Measurement, N (%)	Failed Measurement, N (%)
1st attempt	215 (67.0%)	106 (33.0%)
2nd attempt	59 (69.4%)	26 (30.6%)
3rd or more attempt	22 (75.6%)	7 (24.4%)
**Total**	297	138

## Data Availability

The data presented in this study are available on request from the corresponding author. The data are not publicly available due to privacy and ethical restrictions.

## Abbreviations

The following abbreviations are used in this manuscript:

ABG	Arterial blood gas
CO_2_	Carbon dioxide
TcCO_2_	Transcutaneous carbon dioxide
LTV	Long-term ventilation
NEAVS	North East Assisted Ventilation Service
NIV	Non-invasive ventilation
COPD	Chronic obstructive pulmonary disease
OHS	Obesity hypoventilation syndrome
NMD	Neuromuscular disease
DMD	Duchenne’s muscular dystrophy
SpO_2_	Peripheral oxygen saturation
